# Actinomycin D Arrests Cell Cycle of Hepatocellular Carcinoma Cell Lines and Induces p53-Dependent Cell Death: A Study of the Molecular Mechanism Involved in the Protective Effect of IRS-4

**DOI:** 10.3390/ph14090845

**Published:** 2021-08-25

**Authors:** Luis G. Guijarro, Patricia Sanmartin-Salinas, Eva Pérez-Cuevas, M. Val Toledo-Lobo, Jorge Monserrat, Sofia Zoullas, Miguel A. Sáez, Miguel Angel Álvarez-Mon, Julia Bujan, Fernando Noguerales-Fraguas, Eduardo Arilla-Ferreiro, Melchor Álvarez-Mon, Miguel A. Ortega

**Affiliations:** 1 Unit of Biochemistry and Molecular Biology (CIBEREHD), Department of System Biology, University of Alcalá, 28801 Alcala de Henares, Spain; patricia.sanmartins@uah.es (P.S.-S.); e.p.cuevas@csic.es (E.P.-C.); eduardo.arilla@uah.es (E.A.-F.); 2 Ramón y Cajal Institute of Sanitary Research (IRYCIS), 28034 Madrid, Spain; jorge.monserrat@uah.es (J.M.); maalvarezdemon@icloud.com (M.A.Á.-M.); mjulia.bujan@uah.es (J.B.); mademons@uah.es (M.Á.-M.); 3 Unit of Cell Biology, Department of Biomedicine and Biotechnology, University of Alcala, 28871 Alcala de Henares, Spain; mval.toledo@uah.es; 4 Department of Medicine and Medical Specialities, Faculty of Medicine and Health Sciences, University of Alcalá, 28801 Alcala de Henares, Spain; sofiazoullas@gmail.com (S.Z.); msaega1@oc.mde.es (M.A.S.); 5 Pathological Anatomy Service, Central University Hospital of Defence-UAH Madrid, 28801 Alcala de Henares, Spain; 6 University Center for the Defense of Madrid (CUD-ACD), 28047 Madrid, Spain; 7 Department of Surgery, Medical and Social Sciences, Faculty of Medicine and Health Sciences, University of Alcalá, 28801 Alcala de Henares, Spain; fernando.noguerales@uah.es; 8 Department of General Surgery, Principe de Asturias Hospital, 28871 Alcala de Henares, Spain; 9 Immune System Diseases-Rheumatology, Oncology Service an Internal Medicine, University Hospital Príncipe de Asturias, (CIBEREHD), 28806 Alcala de Henares, Spain; 10 Cancer Registry and Pathology Department, Hospital Universitario Principe de Asturias, 28806 Alcala de Henares, Spain

**Keywords:** nuclear IRS-4, PI3K, hepatocellular carcinoma, β-catenin, cyclin D, pH3, p53

## Abstract

Actinomycin D (ActD) is an FDA-approved NCI oncology drug that specifically targets and downregulates stem cell transcription factors, which leads to a depletion of stem cells within the tumor bulk. Recently, our research group demonstrated the importance of IRS-4 in the development of liver cancer. In this study, we evaluated the protective effects of IRS-4 against ActD. For this study, three hepatocellular carcinoma cell lines (HepG2, Huh7, and Chang cells) were used to study the mechanism of actinomycin D. Most assays were carried out in the Hep G2 cell line, due to the high expression of stem cell biomarkers. We found that ActD caused HepG2 cell necroptosis characterized by DNA fragmentation, decreased mitochondrial membrane potential, cytochrome c depletion, and decreased the levels of reduced glutathione. However, we did not observe a clear increase in apoptosis markers such as annexin V presence, caspase 3 activation, or PARP fragmentation. ActD produced an activation of MAP kinases (ERK, p38, and JNK) and AKT. ActD-induced activation of AKT and MAP kinases produced an activation of the Rb-E2F cascade together with a blockage of cell cycle transitions, due to c-jun depletion. ActD led to the inhibition of pCdK1 and pH3 along with DNA fragmentation resulting in cell cycle arrest and the subsequent activation of p53-dependent cell death in the HepG2 cell line. Only JNK and AKT inhibitors were protective against the effects of ActD. *N*-Acetyl-L-cysteine also had a protective effect as it restored GSH levels. A likely mechanism for this is IRS-4 stimulating GCL-GSH and inhibiting the Brk-CHK1-p53 pathway. The assessment of the IRS-4 in cancer biopsies could be of interest to carry out a personalized treatment with ActD.

## 1. Introduction

In the last decade, hepatocellular carcinomas (HCC) and hepatoblastomas (HB) had global incidences of 400–930 and 1.2–2.5 cases per million per year [1,2], respectively. Notably, both cancers are highly resistant to chemotherapy [3,4]. One of the driving forces of liver cancer relapse post-treatment is the persistence of liver cancer stem cells (LCSCs). LCSCs have been shown to be extremely tumorigenic and resistant to therapy [4]. Despite the large disparity in LCSC characterization, stem cell signatures are maintained in several HCC cell lines including HepG2, HuH7, and Chang cells [5]. HepG2 cells present an upregulation of stem cell transcriptional factors and as a consequence maintain the expression of CD24, CD133, CD44, and SOX 2 [6,7].

Actinomycin D (ActD) is an FDA-approved NCI oncology drug that specifically targets and downregulates the stem cell transcription factor SRY (sex determining region Y)-Box 2 (SOX2), which leads to stem cell depletion within the tumor bulk [8]. Furthermore, ActD has been shown to improve survival in preclinical models of recurrent glioblastoma [9]. Thus, the drug has been useful in the treatment of rare and highly aggressive cancers such as HB [10,11], embryonal sarcoma of the liver, Wilms tumor, rhabdomyosarcoma, Ewing’s disease, and choriocarcinoma [12,13,14]. Recent studies have revealed that ActD is cytotoxic to LCSCs without affecting immortalized normal hepatocytes [15]. This process occurs via the activation of p53 in an AKT-mediated mechanism [16].

Insulin receptor substrate-4 (IRS-4) belongs to the IRS family that is involved in the transmission of signals from the insulin and insulin-like growth factor-1 (IGF-1) receptors to downstream effectors [17]. Additionally, IRS-4 is not able to be inhibited by tyrosine phosphatase SHP-2; thus, it can constitutively activate the AKT pathway [18]. Interestingly, IRS-4 is overexpressed in both benign proliferative lesions, such as uterine leiomyomas [19] and subungual exostosis [20], and malignant diseases, such as breast cancer [21], leukemia [22], lung cancer [23], colorectal cancer [24,25], and liver cancer [26].

The complete sequencing of 7416 [23] and 1220 [27] human cancer genomes revealed the deregulation of IRS-4 gene expression at the transcriptional level, which has been associated with a lower overall survival of patients in several types of cancer [23]. The overexpression of IRS-4 in melanoma [28] has been attributed to a decrease in microRNA-493 (miR-493), which is involved in IRS-4 mRNA destabilization [29]. In gastric cancer, hsa_circ_0023409 accelerates cell growth and metastasis by regulating the IRS-4/PI3K/AKT pathway [30].

In response to the aforementioned discoveries, the present work aims to define the molecular mechanism of ActD in HepG2 cells and to identify the role of IRS-4 in this process.

## 2. Results

### 2.1. Study of the Effect of ActD on the Biochemical and Cellular Parameters of the HepG2 Cell Line

**Figure 1:**
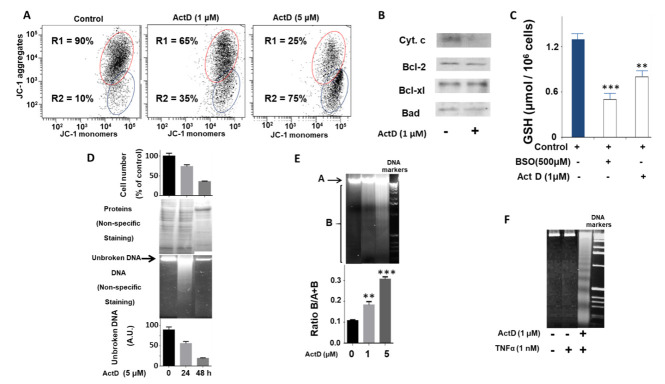
Characterization of the effect of ActD on the biochemical and cellular parameters of the HepG2 cell line. (**A**) Dose-dependent effect of ActD treatment for 24 h on the mitochondrial membrane potential (ΔΨm) monitored by jc-1 dye. Control conditions (5% FBS). The experiment is representative of three others with similar results. (**B**) Cytochrome c, Bcl-2, Bcl-xl, and Bad levels obtained by immunoblot in control conditions (5% FBS) or after ActD (1 µM) treatment for 24 h. The experiment is representative of three others with similar results. (**C**) GSH levels in control conditions (5% FBS) or after ActD (1 µM) treatment for 24 h. As a negative control GSH was measured in the presence of BSO (L-buthionine-(S, R)-sulfoximine) (500 µM) for 24 h. (**D**) Cell number, protein profile, DNA integrity, and evaluation of unbroken DNA of HepG2 cells after Act D (5 µM) treatment at different times. (**E**) Dose-dependent effect of ActD at 24 h of treatment on DNA integrity (upper panel). The bar graph (lower panel) represents the ratio between the fragmented DNA and the total (unbroken plus fragmented) DNA. (**F**) DNA laddering in samples from HepG2 cells treated with Act D (1 μM) and TNF-α (1 nM) for 24 h. Significance levels: ** *p* < 0.01; *** *p* < 0.001.

Firstly, we observed a dose-dependent effect of ActD on the loss of the mitochondrial membrane potential of HepG2 cells incubated for 24 h in the presence of 5% FBS using a JC-1 probe (Figure 1A). The regions R1 and R2 corresponded to cells with normal and damaged mitochondrial membranes, respectively. As the dose of ActD increased, an observable increase in the number of cells in the R2 region also occurred. Next, we evaluated the levels of cytochrome c and various members of the Bcl-2 family (Figure 1B). Incubation of HepG2 cells with ActD (1 µM) for 24 h produced a decrease in cytochrome c levels. There were no apparent changes in the levels of Bcl-2, Bcl-xl, and Bad (Figure 1B).

Furthermore, after the treatment of HepG2 cells with ActD (1 µM) for 24 h, the levels of GSH (Figure 1C) decreased significantly with respect to the positive and negative controls. The negative control (in the presence of an inhibitor of glutathione synthesis) was achieved by treating cells with BSO (L-buthionine-(S, R)-sulfoximine), which specifically inhibits GSH biosynthesis.

Moreover, we observed that ActD produced DNA fragmentation in a time- and dose-dependent manner (Figure 1D,E, respectively). This was evident as the DNA of treated cells was distributed in a smear on the agarose gel (Figure 1D, middle panel). However, when the HepG2 cells were incubated with ActD (1 µM) and TNF-α (1 nM) for 24 h, we observed DNA laddering, which is characteristic of inter-nucleosomal fragmentation (Figure 1F), as previously described [31].

The bar chart of Figure 1D (lower panel) represents the amounts of unbroken DNA obtained by densitometric analysis in absorbance units (A.U.). The cell number at different ActD incubation times is represented in the upper bar chart of Figure 1D. Additionally, the total protein content was visualized using non-specific staining using Coomassie blue (Figure 1D).

Similarly, DNA was fragmented in the presence of increasing concentrations of ActD for 24 h (Figure 1E, upper panel). The quantification of the ratio of A (unbroken DNA) and B (broken DNA) is represented in the histogram of Figure 1E (lower panel).

**Figure 2:**
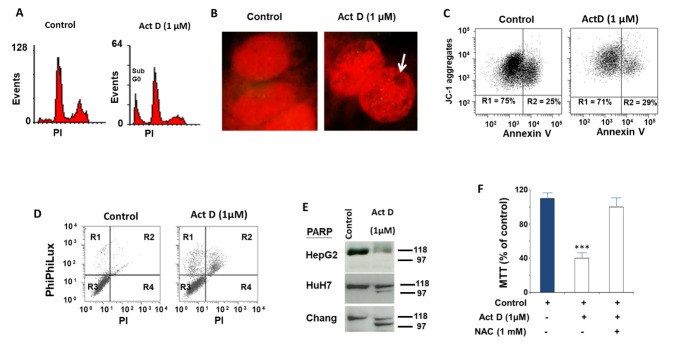
(**A**) Cell cycle analysis by flow cytometry using propidium iodide staining. The sub-G0 population of HepG2 cells treated for 24 h with ActD (1 μM) in the presence of FBS (5%) increased 3-fold with respect to the control cells (5% FBS). The experiment is representative of three others with similar results. (**B**) Microscopic analysis of the nucleus stained with propidium iodide of the HepG2 cell incubated as explained in panel (**A**), 20× (**C**). Study by flow cytometry of the binding of Annexin V to the cell surface of live cells measured using a JC-1 probe. HepG2 cells were incubated as explained in panel (**A**). The experiment is representative of three others with similar results. (**D**) Study of caspase 3 activation by flow cytometry using a PhiPhilux probe. A HepG2 cell incubated as explained in panel (**A**). The experiment is representative of three others with similar results. (**E**) Analysis of PARP proteolysis using western blot in different HCC cell lines treated as explained in panel (**A**). The experiment is representative of three others with similar results. (**F**) Measurement of the MTT reducing ability of HepG2 cells in control conditions (5% FBS), in the presence of NAC (*N*-Acetyl-L-cysteine) (1 mM) plus ActD (1 µM) or with ActD (1 µM) for 24 h. Significance levels: *** *p* < 0.001.

Next, we analyzed cell cycle and the apoptosis of HepG2 cells treated with ActD by flow cytometry and biochemical methods (Figure 2). We detected that ActD treatment (1 µM) of HepG2 cells for 24 h increased the sub-G0 population assessed by propidium iodide (PI) staining (Figure 2A). However, the fluorescent microscope observation of the nuclei stained with PI showed that they had similar sizes to those of the control cells. Although no nuclear fragmentation was observed, chromatin clumping was visualized in several cells (Figure 2B). When we stained the cells with normal mitochondria (viable cells) with Annexin V, we observed that treatment with ActD (1 µM) for 24 h did not increase the number of cells that were positive for Annexin V (Figure 2C). The binding of Annexin V to phosphatidylserine on the cell surface is an early marker of apoptosis, thus suggesting that the effects of ActD in HepG2 cells do not occur through the classical mechanism of apoptotic cell death. In order to further explore this hypothesis, we studied the effect of ActD (1 µM) for 24 h on caspase 3 activity and the PARP proteolysis of HepG2 cells (Figure 2D and Figure 2E, respectively). Using the PhiPhilux probe for monitoring caspase 3 activity (Figure 2D), we observed that ActD treatment increased caspase 3 activity by up to 10% in viable cells (PI negative-R1) and up to 25% in death cells (PI positive-R2). This finding indicates that most cells do not die via apoptosis as classical apoptosis would reflect positive caspase 3 and negative PI.

The activation of caspase 3 correlated with the subsequent proteolysis of PARP; however, we did not observe the classical apoptosis marker of a clear increase in the 97 kDa fragment in HepG2 cells studied under the same conditions. Interestingly, the treatment of HuH7 and Chang cells with ActD (1 µM) for 24 h produced a PARP fragmentation profile compatible with the classical activation of apoptosis (Figure 2E). We decided to continue the study with the HepG2 line because they expressed liver-specific and hepatic cancer stem cells markers such as CD133, alpha fetoprotein, GAPDH, and albumin [6,32]. Following the treatment of HepG2 cells with ActD (1 µM) for 24 h, the reducing capacity of MTT (Figure 2F) decreased significantly with respect to the control cells. The effects of ActD on the MTT assay were reversed by incubation with NAC (*N*-Acetyl-L-cysteine) (1.5 mM), indicating that the effects of ActD on HepG2 cells are largely dependent on GSH levels. On aggregate, the data suggest that ActD induces a GSH-dependent process of cell death in which necroptosis plays an important role.

### 2.2. Effect of ActD on the MAP Kinases and AKT Cascades in HepG2 Cells

**Figure 3:**
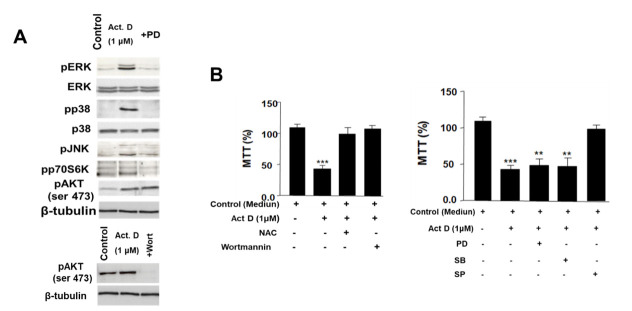
(**A**) Study of the MAP kinases and AKT pathways by immunoblot in HepG2 cells treated with ActD (1 µM) alone or combined with PD98059 or wortmannin for 24 h. (**B**) Effect of different inhibitors on the metabolic activity (MTT) of HepG2 cells during treatment with ActD (1 µM) for 24 h. The experiments from panel (**A**) are representative of three others with similar results. PD = PD98059; Wort = Wortmannin. SP = SP600125; SB = SB203580; NAC = *N*-Acetyl-L-cysteine (1 mM). β-tubulin was used as loading control of SDS-PAGE. Significance levels: ** *p* < 0.01; *** *p* < 0.001.

We observed that ActD (1 µM) incubation for 24 h activated the phosphorylation of MAP kinases (ERK1/2, p38, and JNK) and AKT/P70S6K (Figure 3A). We observed that PD098059 inhibits all the MAP kinases studied as well as P70S6K. This is due to the fact that this drug inhibits the mitogen-activated protein kinase kinase-1 (MAPKK1), which is upstream of the signaling cascade of MAP kinases [33]. However, ActD had an effect on the activation of AKT, which was completely inhibited by the drug wortmannin that acts on PI3K. Therefore, our results suggest that ActD acts on MAPKK1 and AKT through independent mechanisms. To elucidate the signaling pathways involved in the cytotoxic effect of ActD, we co-treated the HepG2 cells with specific kinase inhibitors and with *N*-Acetyl-L-cysteine (NAC) (Figure 3B,C). We observed that wortmannin (PI 3K inhibitor), SP600125 (JNK inhibitor), and NAC were able to reverse the effect of ActD. Thus, the data suggest that the toxic effect of ActD is dependent on the emergence of free radicals and/or GSH depletion and the activation of the JNK and AKT pathways.

### 2.3. Modulation of IRS-4 Expression by ActD in the HepG2 Cell Line

**Figure 4:**
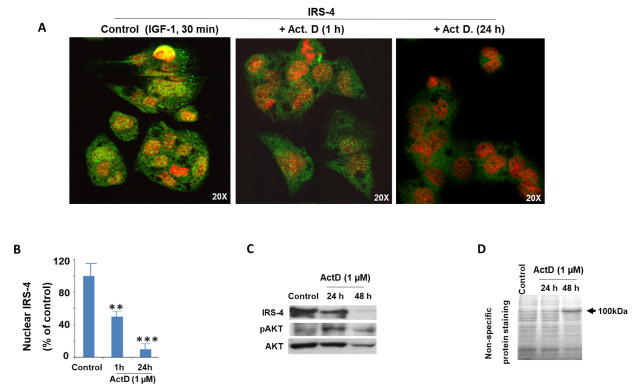
Effect of ActD (1 µM) for different times in IRS-4 expression in HepG2 cells. (**A**) HepG2 cells stained with anti-IRS-4 antibody (green) and propidium iodine (red). For a negative control the anti-IRS-4 antibody was omitted and was only incubated with propidium iodine (data not shown). Cells were incubated with IGF-I (25 nM) for 30 min as described in the Materials and Methods section and after this time ActD was added for 1 h and 24 h. (**B**) Nuclear IRS-4 was assessed from immunocytochemistry experiments as described in the Materials and Methods section. The bar chart represents the mean ± SEM of densitometric intensity of nuclear IRS-4 of four independent experiments. (**C**) Immunoblot of IRS-4 of control HepG2 cells or treated with ActD (1 µM) for 24 and 48 h. This image is representative of 3 others with similar results. (**D**) Protein profile obtained by Coomassie blue staining of the HepG2 cell treated in the conditions described for the panel (**C**). Significance levels: ** *p* < 0.01; *** *p* < 0.001.

Our group observed that IRS-4 plays an important role in cell proliferation and the inhibition of cell death in the HepG2 line [31], so we sought to determine whether ActD treatment affected IRS-4 levels. When we incubated the cells with IGF-1 (25 nM) for 1 h, after a period of 24 h in the absence of 5% FBS, we used confocal fluorescence microscopy to observe an increase in IRS-4 in the nuclei of cells (green staining). However, when we added ActD (1 µM), there was a rapid decrease in IRS-4 levels in all cell compartments (Figure 4A). The nuclei were stained with PI (red staining). In Figure 4B we can observe changes in the optical density of IRS-4 in the nuclei of cells. We corroborated the microscopy results using western blot analyses (Figure 4C). At incubation with ActD for 48 h, we observed the complete degradation of IRS-4 with the total AKT also affected but to a lesser extent; however, pAKT levels remained high (Figure 4C). As a control we visualized the cells using Coomassie Blue, a nonspecific protein stain (Figure 4D). We did not observe changes in the protein profile at 24 h of treatment with ActD; however, important differences were detected at 48 h with the apparition of a 100kDa band (Figure 4D). These data indicate that the decrease in IRS-4 at 24 h of ActD (1 µM) incubation is not due to general protein degradation.

### 2.4. Study of the Protective Role of IRS-4 against ActD in HepG2 Cells

**Figure 5:**
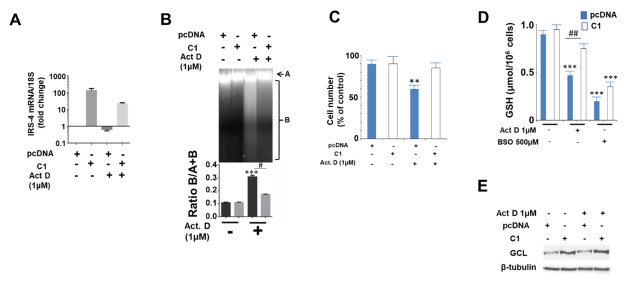
Effect of IRS-4 overexpression on HepG2 cell death induced by ActD. (**A**) IRS-4 mRNA expression levels in C1 and pcDNA colonies obtained from the HepG2 cell line and incubated with Act D (1 µM) for 24 h. (**B**) DNA fragmentation of C1 and pcDNA colonies after treatment with ActD (1 µM) for 24 h. The experiment is representative of three others. (**C**) Number of cells in colonies C1 and pcDNA of HepG2 after treatment with ActD (1 µM) for 24 h. (**D**) Glutathione levels in C1 and pcDNA colonies after treatment with ActD (1 µM) for 24 h. BSO has been used as a control for inhibition because it is a specific competitor of glutamyl cysteinyl ligase (GCL). (**E**) Glutamyl cysteinyl ligase (GLC) levels assessed by immunoblot in C1 and pcDNA colonies after treatment with ActD (1 µM) for 24 h. β-tubulin was used as a loading control of SDS-PAGE. The western blotting experiments are representative of three others with similar results. Significance levels: ** *p* < 0.01; *** *p* < 0.001; ^##^ *p* < 0.01.

Previous research has demonstrated that the stable transfection of pcDNA (IRS-4) increased IRS-4 protein levels in HepG2 cells (C1 line) in comparison to a control group (pcDNA empty vector) [26]. In Figure 5A, we show the levels of IRS-4 mRNA in both C1 and control cells (pcDNA). Treatment with ActD (1 µM) for 24 h decreased IRS-4 mRNA levels in both the C1 and control cells, but this decrease was minimized in the C1 line (Figure 5A).

As demonstrated in Figure 5B, the C1 cell line was more resistant than the control to the effect of ActD (1 µM) for 24 h on DNA integrity, as measured by agarose gel electrophoresis. This effect was correlated with the greater number of C1 cells with respect to control cells following the same incubation conditions with ActD (Figure 5C). The effect of ActD on cell proliferation is GSH-dependent, as we previously demonstrated; thus, we measured GSH concentration (Figure 5D) and glutamyl cysteine ligase (GCL) levels using immunoblot assays. GCL was assessed because it is involved in GSH biosynthesis (Figure 5E). Following ActD treatment, GSH levels remained higher in the C1 colony with respect to the control cells (Figure 5D), which could be explained by the greater GCL levels in the C1 cells with respect to the control that were observed (Figure 5E).

### 2.5. Study of the Mechanisms Involved in the Protective Role of IRS-4 against ActD in HepG2 Cells

**Figure 6:**
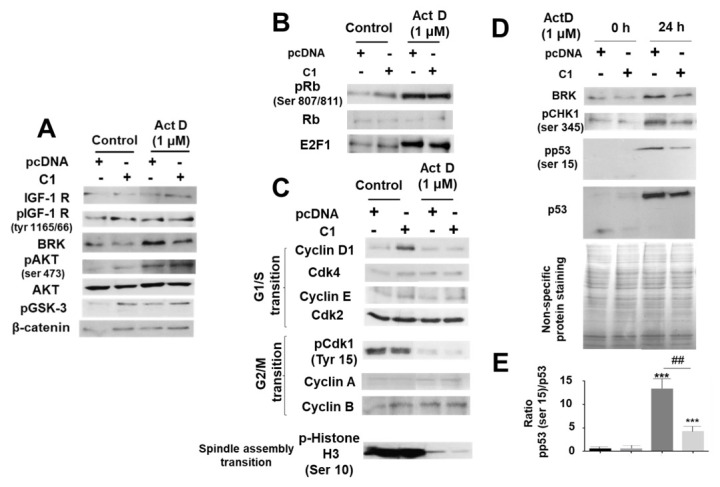
Study of the effect of ActD and IRS-4 overexpression in different regulatory signaling pathways of HepG2 cells. (**A**) Western blot of the IGF-I receptor signaling pathway from C1 and pcDNA HepG2 cells after treatment with ActD (1 µM) for 24 h. (**B**) Western blot of the Rb/E2F signaling pathway from C1 and pcDNA HepG2 cells after treatment with ActD (1 µM) during 24 h. (**C**) Analysis by western blotting of the G1/S and G2/M transitions from C1 and pcDNA HepG2 cells after treatment with ActD (1 µM) for 24 h. (**D**) Western blot of BRK, pCHK1, p53, and pp53 levels from C1 and pcDNA HepG2 cells after treatment with ActD (1 µM) for 24 h. Protein profile obtained by Coomassie blue staining of the HepG2 cell treated in the conditions described for the panel (**D**). (**E**) Bar graph of the results corresponding to the pp53/p53 ratio obtained from the western blot experiments. Experiments from panels (**A**–**D**) are representative of three others with similar results. Significance levels: *** *p* < 0.001; ^##^ *p* < 0.01.

We observed that incubation with ActD (1 µM) for 24 h activated the phosphorylation of AKT and GSK-3 through a mechanism independent from the IGF-1 receptor since we did not detect changes in either the levels of the IGF-1 receptor or in its phosphorylation state (Figure 6A). Interestingly, we did observe an increase in the BRK enzyme in presence of ActD. This enzyme is capable of phosphorylating AKT and directly activating it (Figure 6A). The overexpression of IRS-4 was accompanied by a slight decrease in BRK levels without notable changes in the IGF-1R-AKT-GSK-β-catenin system (Figure 6A). The activation of AKT following ActD (1 M) incubation for 24 h was accompanied by an increase in pRb (ser 807/811) and E2F (Figure 6B). However, biomarkers for the G1/S transition (Cyclin D-Cdk4, Cyclin E-Cdk2) did not increase and biomarkers for the G2/M transition, such as pCdk1 (Tyr 15) and pH3 (ser 10), were markedly decreased compared to the control (Figure 6C). In addition, we observed an increase in pCHK1 (ser 345), pp53, and p53 following ActD (1 µM) treatment for 24 h (Figure 6D). These results suggest a blockage of the cell cycle in G2/M phase induced by ActD, consequently generating an increase in the BRK-pCHK1-p53 cascade (Figure 6D). Following ActD treatment, p53 levels increased, both in relation to its phosphorylation and in quantity (Figure 6D). The total amount of protein stained with Coomassie Blue was used as a charge control for the electrophoresis (Figure 6D, lower panel). In Figure 6E, we show the effects of ActD on the phosphorylated p53/total protein level ratio. As we can see in the different panels of Figure 6, the overexpression of IRS-4 (colony C1) did not produce appreciable changes following the ActD treatment either in the signaling cascade (Figure 6A) or in the Rb-E2F cascade (Figure 6B), or in the enzymes involved in the control of G1/S and G2/S transitions (Figure 6C). However, we did observe a consistent decrease in BRK-pCHK1 and pp53/p53 cascades in C1 cells with respect to the control cells following ActD treatment (Figure 6D,E).

### 2.6. Study of the Effect of IRS-4 Downregulation and ActD Treatment on Gene Expression in HepG2 Cells

**Figure 7:**
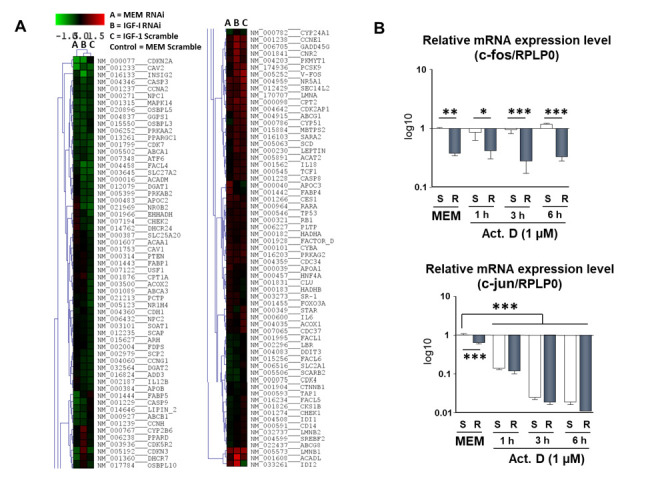
Microarray gene expression profile of HepG2 cells. (**A**) Hierarchical clustering analysis of the gene expression profiles of HepG2 cells corresponding to the following groups: MEM RNAi (IRS-4 mRNA levels were decreased by knockdown and maintained for 24 h in MEM); IGF-1 RNAi (IRS-4 mRNA levels were decreased by knockdown and maintained for 24 h in IGF-1 (25 nM)); IGF-1 scramble (mRNA levels from HepG2 cells treated with scrambled oligos and maintained for 24 h in IGF-1 (25 nM)). The expression levels of the mRNA from the three groups were compared with the control (mRNA levels from HepG2 cells treated with scrambled oligos and maintained for 24 h in MEM). The experiment is representative of two others with similar results. (**B**) Relative mRNA expression levels of c-fos and c-jun obtained by a qPCR in the HepG2 cells in which IRS-4 mRNA levels were decreased by knockdown (R) or were treated with scrambled oligos (S). HepG2 cells were maintained in MEM or in the presence of ActD (1 µM) for 1, 3, or 6 h. RPLP0 was used as control of expression. Significance levels: * *p* < 0.05; ** *p* < 0.01; *** *p* < 0.001.

We studied the mRNA expression of 123 genes by microarray in the IRS-4 mRNA knockdown of HepG2 cells (Figure 7A).

The genes analyzed were grouped into eight major functional groups: apoptotic (p53, caspase-3, -8, -9), cell cycle regulators (CDKN2A, CDK5R2, CDKN3, RB1, CDK4, CCDK5R2, CDK7, CDK2AP1), signaling (PTEN, MAPK14), metabolic (APOC2, APOB, CAV1, CAV2), trans regulatory factors (Insig2, SREBF2, ATF6), transporters (ABCA1, ABCA3, ABCB1, ABCG8), cytokines (IL6, IL18), and cytochromes (CYP2B6). Four experiments were performed: (1) MEM Scramble (control group), in which cells were treated by scramble and cells were incubated with MEM for 24 h; (2) MEM RNAi group, in which IRS-4 expression was suppressed, as described in the Materials and Methods section, and cells were incubated in MEM; (3) IGF-1 scramble group, in which cells were treated by scramble RNA and then treated with IGF-1 (25 nM) for 24 h; (4) IGF-1 RNAi group, in which IRS-4 expression was suppressed and cells were treated with IGF-1 (25 nM) for 24 h.

The most relevant and consistent result was that the decrease in IRS-4 levels in HepG2 cells led to a decrease in CDKN2A gene expression, both in the presence and absence of IGF-1 with respect to the control (Figure 7A). We observed no difference in the expression of previously studied genes, such as Cdk4, Rb, and p53, which is consistent with the western blot results obtained from cells overexpressing IRS-4 (Figure 6B–D). At the transcriptional level, CDKN2A is strongly regulated by the AP-1 (activator protein-1) complex. For this reason, we studied the mRNA levels of c-fos and c-jun in HepG2 IRS-4 knockdown cells in the absence or presence of ActD (1 µM) for different periods of time. The results are shown in Figure 7B.

The decrease in IRS-4 levels by siRNA (S) resulted in a significant decrease in c-fos and c-jun mRNA compared to the control cells (S). Treatment with ActD (1 µM) for different time periods (1–6 h) did not change the c-fos levels but caused a profound decrease in c-jun mRNA in a time-dependent manner (Figure 7B).

## 3. Discussion

Actinomycin D is attracting the attention of pharmacologists because it is able to inhibit stem cells present in the most aggressive tumors, such as glioblastomas [9], breast cancer [8], or liver cancer in a very efficient way [15]. Furthermore, ActD inhibits the activity of liver cancer stem cells (LCSCs) without affecting healthy, co-cultured hepatocytes [15]. We used the HuH7, Chang, and HepG2 cell lines to study this phenomenon in an attempt to elucidate the molecular mechanisms involved. The majority of experiments were carried out using the HepG2 cell line since it expresses a high proportion of stem cell biomarkers such as CD133, alpha fetoprotein, GAPDH, and albumin [6,32]. Furthermore, we have shown that ActD induces cell death in this cell line, thus HepG2 cells provide a good model for studying the cytostatic effect of ActD on liver-specific stem cells.

**Figure 8:**
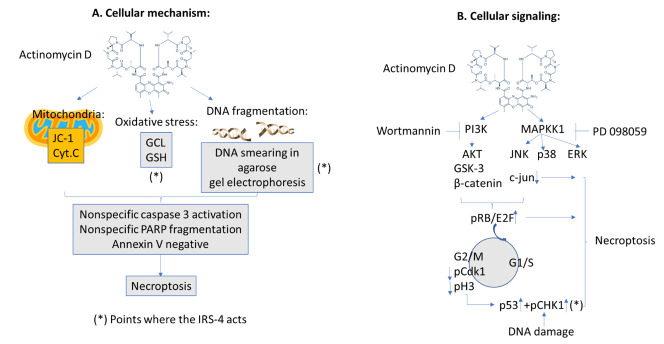
Schematic model of the results obtained in the present work. (**A**) Cellular mechanism of the work. (**B**) Cellular signaling of the work.

We believe that the effect of ActD on HepG2 cells is better defined by necroptosis than apoptosis because we did not observe a clear increase in annexin V or in bcl-2, bcl-xl, and bax levels following treatment with the drug. Likewise, we observed a smear DNA fragmentation with a slight activation of caspase 3, which gave rise to a non-specific fragmentation of PARP. However, in HuH7 and Chang cells, ActD produced a PARP fragmentation compatible with apoptosis. In HepG2 cells, ActD induced a decrease in cytochrome c and GSH levels which led to a loss of mitochondrial membrane potential, so the cells were unable to enter into apoptosis due to a lack of energy, resulting in necroptosis (Figure 8). When we combined ActD with TNFα, an extrinsic apoptotic process in HepG2 cells was activated, in accordance with previous research [11]. At present, we do not know what the causes of the differences in the effect of ActD on liver cells HepG2, HuH7 and Chang liver cell are. In recent articles, the latter line has not been considered as a suitable model due to the suspicion that they may be contaminated by Hela cells. In relation to the other two HCC cellular lines, it has been described that the effects of sorafenib, which belongs to the tyrosine kinase inhibitors family, depends on several factors such as cell differentiation, mitochondrial respiration, and p53 status [34]. Relative to the latter molecule, HepG2 cells harbor wild type p53 and Huh7 has a point mutation in p53 [34] which could be one of the possible explanations of the observed effects.

Signaling studies show that ActD-induced cell death is due to the activation of PI3K and MAPKK1 since wortmannin is able to inhibit the effect of the drug on pAKT, and PD098059 is able to inhibit pp38, pERK, and pJNK, which are downstream in the MAPK pathway [33,35]. The two cascades that appear to be related to the effects of ActD on cell death are AKT and JNK, because wortmannin and SP600125 inhibited the effects of ActD on necroptosis. Curiously, we observed a significant decrease in c-jun expression together with JNK activation. Although JNK stimulation is a general mechanism of apoptosis initiation because it stimulates the expression of pro-apoptotic genes through c-jun phosphorylation, the stark reduction in c-jun could preclude apoptosis. Moreover, it has recently been shown that c-jun regulates the efficient transition of the G1/S cell cycle phase and cells lacking c-jun have a severe proliferation defect [36]. Moreover, research has shown that a decrease in c-jun could arrest cell cycle [37].

It should also be noted that oxidative stress plays an important role in ActD-induced necroptosis of hepatoblastoma cells because treatment with NAC reverses this process. The predominant cascade involved in ActD-induced cell death is unknown, but we observed the stimulation of the RB/E2F signaling cascade.

However, this was not reflected in surpassing the G1/S and G2/M checkpoints. This can be explained by the decrease in c-jun at the first checkpoint and the decrease in pCdK1 and pH3 at the second one, which is indicative of a blockage in the cell cycle at different levels. The activation of a p53-dependent cell death process is a well-known consequence of cell cycle blockage [38]. In addition, DNA fragmentation leads to the activation of pCHK1 [39]. Thus, the observed activation of the pCHK1-p53 cascade in conjunction with the increase in E2F and decrease in c-jun caused the necroptosis of hepatoblastoma cells (Figure 8). In other cellular models, E2F in the presence of damaged DNA could contribute to apoptosis induction via cooperation with p53 [40]. Interestingly, the treatment of HepG2 cells with ActD, even for short periods of time (1 h), caused a decrease in IRS-4 levels, mainly in the nucleus of the cells as we can see in the IHC experiments. We must bear in mind that ActD is a powerful transcription inhibitor [41] that acts very quickly, as we have been able to verify when studying the levels of c-jun mRNA. We cannot rule out that the ActD-induced depletion of IRS-4 is the main cause of the drug’s effects on proliferation; however, the fact that IRS-4 overexpression does not protect from the effects of ActD on the protein regulators of G2/M (pCdk1) and spindle assembly (pH3) transitions, makes us think that it is unlikely.

In the studied hepatoblastoma model, the overexpression of IRS-4 protected against the effects of ActD because it favored the synthesis of GCL, causing an increase in GSH levels, a decrease in the pCHK1-p53 cascade, and a decrease in DNA fragmentation. However, the increase in IRS-4 did not prevent ActD-induced c-jun depletion. At the present time, we do not know if the increase in GCL-GSH and the decrease in pCHK-p53 in cells that overexpress IRS-4 are related. It could be that the regulation of GSH levels by IRS-4 is the connection between both processes because an earlier study in HepG2 cells showed that fine control of the GSH levels is essential for translating ROS signals into a p53-dependent apoptosis pathway [42]. Therefore, this is a hypothesis for future studies.

At present, the excellent efficacy of ActD in the control of stem cells is not well understood, but it is thought to be mediated by DNA binding in regions rich in G-quadruplex, which are abundant in CpG islands, microsatellites, and telomeric repeats [43,44]. Telomerase enzyme is upregulated in stem cells and in ~85% of cancers cells, but not in somatic cells [45]. In our study, we used ActD in the micromolar concentration range because previous pharmacokinetic studies showed that it accumulates in the liver at high concentrations in from 500 to 2500 μg/L [46]. These concentrations are compatible with the concentrations used in present study.

It is probable that the mechanisms discussed above explain why ActD is the first line of treatment for different types of choriocarcinomas [14] and why it is useful in the therapy of rhabdomyosarcoma, Wilms tumor, Ewing’s sarcoma [12,13], and in the neoadjuvant therapy of hepatoblastoma [10,11]. The potential for effective clinical applications of this well-known drug reiterates the importance of research into its mechanism of action and the importance of identifying the genes that could provide resistance to it.

## 4. Materials and Methods

### 4.1. Cell Culture and Incubation Conditions

The human HB cell lines HepG2, HuH7, and Chang cells were obtained from ATCC and maintained in MEM (Gibco, Grand Island, NY, USA) or supplemented with 5% fetal bovine serum (FBS) and 1% antibiotic/antimycotic solution at 37 °C in a 5% CO_2_ humidified incubator, as previously described [47].

HepG2 cells were incubated with ActD (1 μM or 5 μM) for different times (1 to 48 h) in the absence or in the presence of wortmannin (200 nM), PD098059 (30 µM), SP600125 (30 µM), SB203580 (30 µM), or *N*-Acetyl-L-cysteine (NAC) (1.5 mM).

To study the stimulatory effect of IGF-1, HepG2 cells were starved for 72 h and then stimulated with IGF-1 (25 nM) for 30 min. Cells were then studied by immunocytochemistry or lysed for further biochemical analysis, as previously described [48]. In microarray experiments, HepG2 cells were transfected with siRNA oligos and incubated with IGF-1 (25 nM) for 24 h.

### 4.2. Transfection Assays

HepG2 cells were transfected with pcDNA (IRS-4) or the empty vector (pcDNA), as previously described [25] with minor modifications. Briefly, full-length IRS-4 DNA (3881 bp) isolated from RKO cells was amplified by a nested PCR using Pwo DNA polymerase (Roche). The DNA products were ligated at the HindIII and EcoR1 sites of pcDNA3.1. Then, the recombinant plasmid was assessed by DNA sequencing.

The transfection of HepG2 cells with pcDNA3.1-IRS-4 was performed using TurboFect (Thermo Scientific, Waltham, MA, USA) according to the manufacturer’s instructions. The empty pcDNA3.1 vector was used to obtain the corresponding control (hereinafter pcDNA). Stable transfectants were obtained after selection with G418 for different periods of time. Several colonies overexpressing IRS-4 were obtained [26]. For further studies, the colony C1 was selected.

Regarding the transfection with siRNA oligos, the SiRNA oligos corresponded to nts 819–839 of the human IRS-4. SiRNA and scrambled oligos were synthesized by Dharmacon Research Inc. The transfection of the HepG2 cells was performed in the presence of oligofectamine in a final volume of 1 mL of OPTIMEM, according to the manufacturer’s instructions (Invitrogen, Waltham, MA, USA).

Cells were maintained at 37 °C for different periods of time (48 to 72 h) with IRS-4 siRNA or scrambled oligos. Cells were lysed for further analysis following ActD treatment.

### 4.3. Immunocytochemistry

HepG2 cells were seeded onto glass coverslips, grown in DMEM containing 5% FBS. After 72 h of serum starvation, HepG2 cells were stimulated with IGF-1 (25 nM) for 30 min, then ActD (1µM) was added, and incubation continued for 1 h and 24 h. Coverslips were immersed in 4% paraformaldehyde, washed in PBS, permeabilized with 0.05% Triton X-100, and blocked for 10 min with donkey serum (3%). Cells were then incubated with an anti-IRS-4 IgG antibody (Upstate Biotechnology, Lake Placid, NY, USA) followed by chicken anti-rabbit-Alexa Fluor 488. Coverslips were mounted using FluoroshieldTM with propidium iodine (Sigma-Aldrich, Madrid, Spain) and visualized using Leica TSC-SL confocal microscopy [48].

### 4.4. Biochemical and Molecular Biology Methods

Protein was extracted from the HepG2 cells as previously described [31]. HepG2 cell lysates (40 μg of protein) were analyzed by SDS-PAGE and western blot assays, as previously described [49]. Intracellular GSH levels, MTT reduction assays, and DNA fragmentation gel assays were conducted according to the methods described previously [47,50,51].

Gene expression studies were carried out using microarrays following the procedure that has been extensively described previously [52]. The sequence of the cDNA probes was obtained from the consortium IMAGE database and Open Biosystems. The cDNA samples from all clones were amplified by a PCR using universal primers and the quality of the products was tested using agarose gel electrophoresis and sequencing. Microarrays containing human cDNA probes for 123 genes were produced using a SpotArray 72 spotter (PerkinElmer, Waltham, MA, USA).

Total RNA extraction and Poly(A)+ RNA purification from HepG2 cells was performed using the TriReagent (Sigma, St. Louis, MO, USA) and GenElute mRNA Miniprep Kit (Sigma), respectively, according to the manufacturer’s protocols. The mRNA was labeled with cyanine 3 or cyanine 5 labeling reagents using the MICROMAX™ ASAP RNA Labeling Kit (PerkinElmer, Waltham, MA, USA), according to the manufacturer’s instructions. Each sample was evaluated at 3–6 replicate points, averaged, and transformed in log 2 using the Gene Expression Pattern Analysis Suite v3.1 (GEPAS) preprocessing tool. The data was clustered and visualized using Mev v3.1 software.

For qPCR experiments, the total RNA was isolated using RNeasy Mini Kit (Qiagen, Hilden, Germany) according to the manufacturer’s protocol. Contaminating genomic DNA was eliminated using RNase-free DNase (Qiagen). Total RNA (2 μg) was reverse transcribed into single-stranded cDNA using the AMV First Strand cDNA synthesis kit (Roche, Basilea, Switzerland), according to the manufacturer’s instructions. Real-time PCR amplification reactions were performed using the SYBR Green PCRMaster Mix (Applied Biosystems, Waltham, MA, USA). The cycling conditions and the primers used to amplify IRS-4, 18S, c-fos, c-jun, and RPLP0 have been previously described [24,53].

### 4.5. Cytometry: Mitochondrial Activity, Apoptosis, and Cell Cycle

After the incubation of HepG2 cells with ActD (1 μM or 5 μM) for 24 or 48 h, adhered cells were collected by trypsinization and washed in PBS. Mitochondrial membrane potential assessment, apoptotic cell number determination, and caspase 3 activation were determined by flow cytometry as previously described with minor modifications [47]. The HepG2 cells were incubated during 24 or 48 h with ActD (1 μM or 5 μM). Then the adhered cells were collected by trypsinization and washed in PBS. Mitochondrial membrane potential loss was assessed using a JC-1 probe (5,50,6,60-tetrachloro-1,10,3,30 tetra-ethylbenzimidazolyl carbocyanine iodide/chloride; Mito-screen, BD Bioscience, San Jose, CA, USA) as previously described [47]. Simultaneously, we identified apoptotic cells by labeling with an Annexin V stain conjugated to allophycocyanin (APC). In the other set of experiments, apoptotic cells were determined by flow cytometry detection of their sub-G0 DNA contents using propidium iodine (PI). Samples were analyzed in a FacsAria cytometer (Becton Dickinson, San Jose, CA, USA) using FacsDiva software. In the study of caspase 3 activation, we used the Phiphilux method following the manufacturer’s instructions. Cells were incubated with 120 µL of the Apo-ONE^®^ Caspase-3/7 Assay Reagent (Promega corporation, Madison, WI, USA) and propidium iodine for 1 h. After this time, samples were analyzed in a FacsAria cytometer (Becton Dickinson, San Jose, CA, USA) using FacsDiva Software 6.0.

### 4.6. Statistical Analysis

The statistical differences of the cellular and biochemical parameters studied (percentage of stained nuclei, cell number, damaged DNA, mRNA expression, MTT and GSH levels) between the subgroups were analyzed by an ANOVA test with a Bonferroni correction. At least three independent experiments were performed to obtain each result. The data are expressed as mean ± SEM. The levels of significance were set at *p* < 0.05 (*), *p* < 0.01 (**) and *p* < 0.001 (***).

## 5. Conclusions

In conclusion, this study unraveled a part of ActD’s mechanism of action in HepG2 cells, which could be a very useful drug in the control of liver cancer stem cells. This is an important step in applying a medication in a personalized way. Furthermore, we showed that IRS-4, an embryonic expression gene, protects against the effects of ActD in HepG2 cells.

## Data Availability

Data is contained within the article.
